# Plasma neurofilament light chain and glial fibrillary acidic protein in the differential diagnosis of acute vertigo in the emergency department

**DOI:** 10.1007/s00415-026-13799-w

**Published:** 2026-04-10

**Authors:** Melanie Haidegger, Natalie Berger, Simon Fandler-Höfler, Lukas Gattermeyer-Kell, Isra Hatab, Markus Kneihsl, Sebastian Monsberger, Tobias Niedrist, Cansu Tafrali, Maria Martinez-Serrat, Pascal Benkert, Jens Kuhle, Christian Enzinger, Michael Khalil, Thomas Gattringer

**Affiliations:** 1 Department of Neurology, Medical University of Graz, Graz, Austria; 2 Division of Neuroradiology, Vascular and Interventional Radiology, Department of Radiology, Medical University of Graz, Graz, Austria; 3 Clinical Institute of Medical and Chemical Laboratory Diagnostics, Medical University of Graz, Graz, Austria; 4 Multiple Sclerosis Centre and Research Center for Clinical Neuroimmunology and Neuroscience (RC2NB), Neurology, Departments of Biomedicine and Clinical Research, University Hospital and University of Basel, Basel, Switzerland

**Keywords:** Vertigo, Neurofilament light chain, Glial acidic fibrillary protein, Blood biomarker, Stroke

## Abstract

**Background:**

Distinguishing posterior circulation stroke (PCS) from peripheral or other causes of acute vertigo can be challenging. Blood biomarkers, such as plasma neurofilament light chain (pNfL) and glial acidic fibrillary protein (pGFAP), have been associated with (even small) stroke lesions and could support patient selection for neuroimaging and inpatient evaluation in acute vertigo.

**Methods:**

This prospective study included consecutive patients presenting with acute vertigo to a neurological emergency department. All patients received neurological examination, laboratory testing and neuroimaging. pNfL and pGFAP were measured using Single Molecule Array (SIMOA) technique. pNfL and pGFAP levels were compared between PCS and patients with non-central vertigo.

**Results:**

102 patients were included (mean age 57.9 years, 57.8% female). pNfL Z-scores were higher in PCS compared to non-central vertigo patients (median 2.1, IQR = 1.5 versus 0.7, IQR = 1.9; *p* = 0.004). pNfL Z-score ≥ 1.0 was associated with PCS (OR 8.8, 95% CI 2.4–32.7), yielding a sensitivity of 84.2%, specificity of 62.4% and negative predictive value of 94.6%. pGFAP Z-scores were higher in PCS patients than in non-central vertigo patients (median 1.3, IQR = 2.8 versus 0.6, IQR = 1.5; *p* = 0.016). pGFAP Z-score ≥ 1.0 was associated with PCS (OR 3.5, 95% CI 1.3–9.8), yielding a sensitivity of 57.9%, specificity of 71.8% and negative predictive value of 88.4%. Respective associations remained robust after correcting for symptom duration.

**Discussion:**

pNfL and pGFAP levels were higher in PCS compared to patients with non-central causes of vertigo. With its high negative predictive value, pNfL appears especially clinically useful to rule out PCS in acute vertigo.

## Introduction

Vertigo, or dizziness, is one of the most frequent reasons for presentation to a neurological or interdisciplinary emergency department. It is important to distinguish between central and more benign peripheral or other causes of vertigo, as central causes, such as posterior circulation stroke (PCS), can cause severe disability and life-threatening complications. However, distinguishing between PCS and other, non-central causes of vertigo can be challenging in clinical practice. Patients often present with heterogeneous complaints and clinical tests can be non-specific [1, 2]. While the sensitivity of widely available computed tomography (CT) for ruling out PCS is low, magnetic resonance imaging (MRI) is time- and resource-consuming and not always available. Furthermore, even diffusion-weighted imaging (DWI) MRI can be negative in acute PCS, particularly when small areas in the brainstem are affected [3].

Considering these limitations, there is an increasing demand for additional diagnostic pathways to support clinical decision making in acute vertigo, especially in determining which patients require (advanced) neuro- and vascular imaging and inpatient evaluation. To date, only few studies have focused on blood biomarkers to distinguish PCS from other causes of non-central acute vertigo. Investigated parameters included brain-specific parameters, such as S100 calcium binding protein beta (S100beta) or neuron-specific enolase (NSE) [4–6]. Further promising biomarkers that have been associated with ischemic stroke and intracranial hemorrhage are neurofilament light chain (NfL) and glial fibrillary acidic protein (GFAP) [7–10]. NfL is a biomarker for axonal damage as it is part of the cytoskeleton of neurons. GFAP is a brain-specific protein that is primarily found in astrocytes. Both biomarkers have been associated with early brain tissue damage, even in small ischemic stroke lesions [11, 12]. However, these studies did not focus on infratentorial locations of stroke lesions specifically. Recently developed ultrasensitive analysis methods using Single Molecule Array (SIMOA) technology enable precise quantification of NfL and GFAP in blood plasma.

Therefore, the aim of this pilot study was to investigate plasma levels of NfL (pNfL) and GFAP (pGFAP) in patients presenting with acute vertigo to the emergency department. We hypothesized that pNfL and pGFAP were elevated in patients with PCS and might support further clinical decision making in this patient cohort.

## Methods

### Patient selection and clinical presentation

**Fig. 1:**
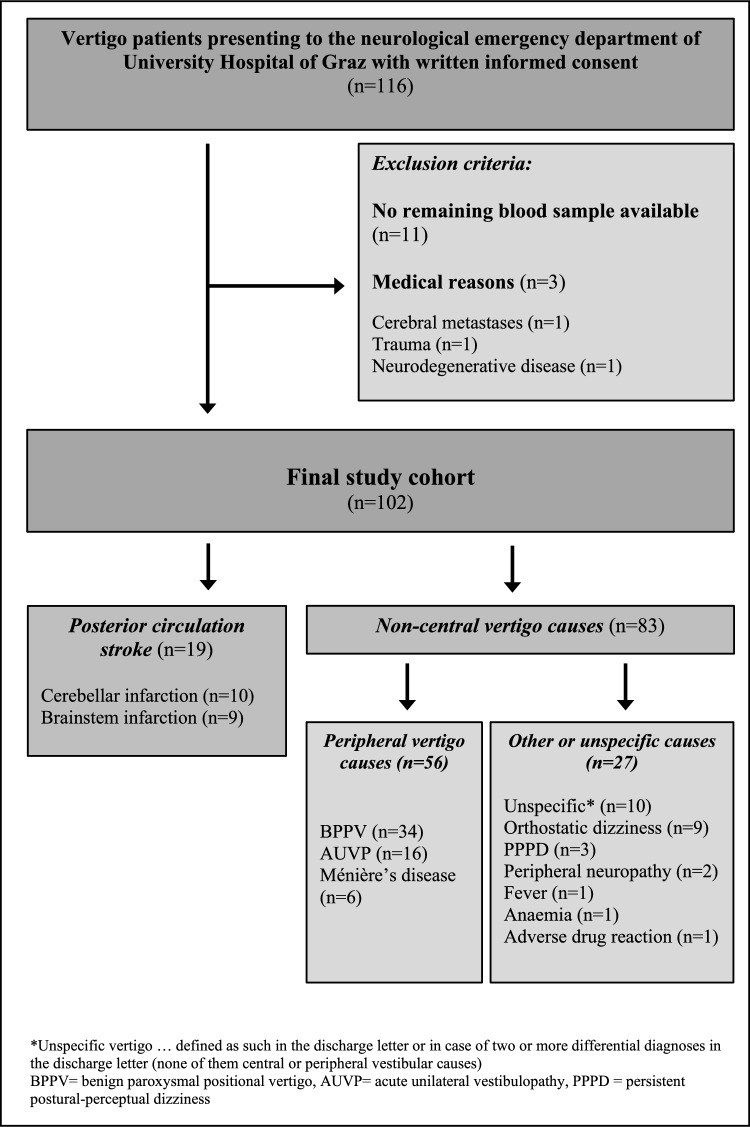
Flow chart of patient selection

This study was designed as a prospective, observational study. Consecutive patients older than 18 years who presented with acute vertigo to the neurological emergency department within the interdisciplinary emergency centre of the University Hospital of Graz from June to December 2024 and gave written informed consent were included. Peripheral blood was collected as part of the routine clinical procedures upon admission in the emergency department. Study-specific laboratory analyses (pNfL, pGFAP) were conducted using the remaining blood from these routinely drawn blood samples. Therefore, no additional venepunctures were performed for study-specific purposes. Patients without available residual blood samples were excluded (Fig. 1). The remaining blood samples were then frozen and stored according to international consensus guidelines [13]. The analysis of pNfL and pGFAP was performed retrospectively. Therefore, the measurements had no influence on further diagnostic work-up and treatment of the included patients.

All patients received clinical neurological assessment and brain imaging on the day of presentation to the emergency department (CT or brain MRI). Further diagnostic tests, including advanced cerebral imaging, neurovascular ultrasound or consultations, with other medical specialties (e.g., otorhinolaryngology, including videonystagmography, video head impulse testing and audiometry, or internal medicine) were performed according to the clinical presentation as indicated by the treating neurologists. Clinical data including the final diagnosis, concomitant diseases, vascular risk factors, current medication, further laboratory parameters of interest, imaging data and the further clinical course of the patient (inpatient admission or discharge) were obtained from the hospital electronic medical documentation system (MEDOCS), which captures clinical data of all 11 public hospitals in the wider catchment area of the province of Styria, covering over 95% of all acute diseases treated in a hospital in this region.

According to the final diagnosis, the patient cohort was divided in two subgroups: PCS and other non-central vertigo causes. The subgroup of non-central vertigo patients included patients with peripheral vertigo causes (acute unilateral vestibulopathy, Ménière’s disease or benign paroxysmal positional vertigo) and other or unspecific vertigo causes. The final diagnoses of the non-central vertigo cohort are presented in Fig. 1.

### Blood biomarker assessment

Peripheral blood samples were taken on the day of presentation in the emergency department. These samples were then centrifuged and stored at −80 °C [13]. pNfL and pGFAP were measured using the Neurology-2-plex B assay on the SIMOA HD-X analyzer (Quanterix, Billerica, MA) according to the manufacturer’s instructions. Furthermore, Z-scores for pNfL and pGFAP were calculated. These Z-scores based on large healthy cohorts were corrected for age and BMI (and in addition sex for pGFAP) [14, 15].

### Statistics

For statistical analyses, the software IBM SPSS (Version 29) was used. Continuous variables were presented as mean and standard deviation or as median and interquartile range; nominal variables were shown in absolute numbers and percentages. Pearson’s Chi-square test was used to compare nominal data. For the comparison of continuous data, Student’s t test or Mann–Whitney U Test was used. Gaussian distribution was assessed with the Kolmogorov–Smirnov test.

A two-way ANOVA was performed to evaluate potential interaction effects between the factors “symptom onset” (below or above 24 h) and “cause of vertigo” (PCS versus non-central cause) on total plasma levels and Z-scores of pNfL and pGFAP.

C-statistic (Receiver Operating Characteristic (ROC) curve analysis) was used to evaluate the sensitivity, specificity and negative predictive values of different pNfL and pGFAP Z-score cut-off levels for identifying PCS. Multinomial logistic regression was used to adjust for symptom onset.

Statistical significance was defined as a probability value below 0.05.

## Results

The final study cohort comprised 102 patients (mean age 57.9 years, 57.8% female). PCS was identified in 19 patients (18.6%), a peripheral vestibular cause in 56 patients (54.9%) and other causes in 27 patients (26.5%). An overview on the final diagnoses is presented in Fig. 1.

Patients with PCS were older (66.7 versus 55.9 years), more often male (62.8 versus 56.3%) and more frequently had common vascular risk factors. In addition, PCS patients presented more often within 24 h after symptom onset to the emergency department in comparison to patients with non-central vertigo causes (Table 1).

**Table 1:** Demographic and clinical characteristics of the patient cohort

	All (*n* = 102)	Central vertigo (*n* = 19)	Non-central vertigo (*n* = 83)	*P* value
Age (years)	57.9 (± 16.5)	66.7 (± 11.4)	55.9 (± 16.9)	0.009
Female sex (%)	59 (57.8)	7 (36.8)	52 (62.7)	0.040
Characteristics of Vertigo (*n*, %)				
Continuous vertigo	50 (49.0)	17 (89.5)	33 (39.8)	< 0.001
Episodic vertigo	52 (51.0)	2 (10.5)	50 (60.2)	< 0.001
Symptom onset < 24 h	69 (67.6)	18 (94.7)	51 (67.6)	0.005
Onset to presentation in ED (days, median, IQR))	0.0 (3)	0.0 (0)	1.0 (4)	0.004
Overall Neuroimaging				
Computed tomography	80 (78.4)	13 (68.4)	67 (80.7)	0.240
Magnetic resonance imaging	46 (45.1)	18 (94.7)	28 (33.7)	< 0.001
Neuroimaging within 24 h				
Computed tomography	79 (77.5)	12 (63.2)	67 (80.7)	0.098
Magnetic resonance imaging	24 (23.5)	13 (68.4)	11 (13.3)	< 0.001
Vascular risk factors (*n*, %)				
Arterial hypertension	45 (44.1)	14 (73.7)	31 (37.3)	0.004
Diabetes	19 (18.6)	9 (47.4)	10 (12.0)	< 0.001
Hyperlipidaemia	36 (35.3)	17 (89.5)	19 (22.9)	< 0.001
Active smoking	21 (20.6)	7 (36.8)	14 (16.9)	0.043
Coronary artery disease	12 (11.8)	5 (26.3)	7 (8.4)	0.029
Atrial fibrillation	10 (9.8)	4 (21.1)	6 (7.2)	0.068
Previous Stroke	9 (8.8)	2 (10.5)	7 (8.4)	0.772
Medication at presentation in the emergency department (*n*, %)				
Antihypertensives	42 (41.2)	13 (68.4)	29 (34.9)	0.007
Antiplatelets	21 (20.6)	6 (31.6)	15 (18.1)	0.189
Oral anticoagulation	9 (8.8)	4 (21.1)	5 (6.0)	0.037
Statins	29 (28.7)	8 (42.1)	21 (25.6)	0.152
Antidiabetics	16 (15.7)	7 (36.8)	9 (10.8)	0.005
Psychotropic medication	23 (22.5)	2 (10.5)	21 (25.3)	0.164
Blood biomarker (median, IQR)				
pNfL (pg/mL)	14.8 (19.4)	21.0 (24.3)	12.4 (15.9)	0.001
pNfL Z-score	0.9 (2.0)	2.09 (1.53)	0.70 (1.88)	0.004
pGFAP (pg/mL)	121.5 (130.7)	211.9 (792.8)	116.8 (99.3)	< 0.001
pGFAP Z-score	0.6 (1.6)	1.34 (2.81)	0.55 (1.53)	0.016

*ED* emergency department, *pGFAP* plasma glial fibrillary acidic protein, *pNfL* plasma neurofilament light chain

**Fig. 2:**
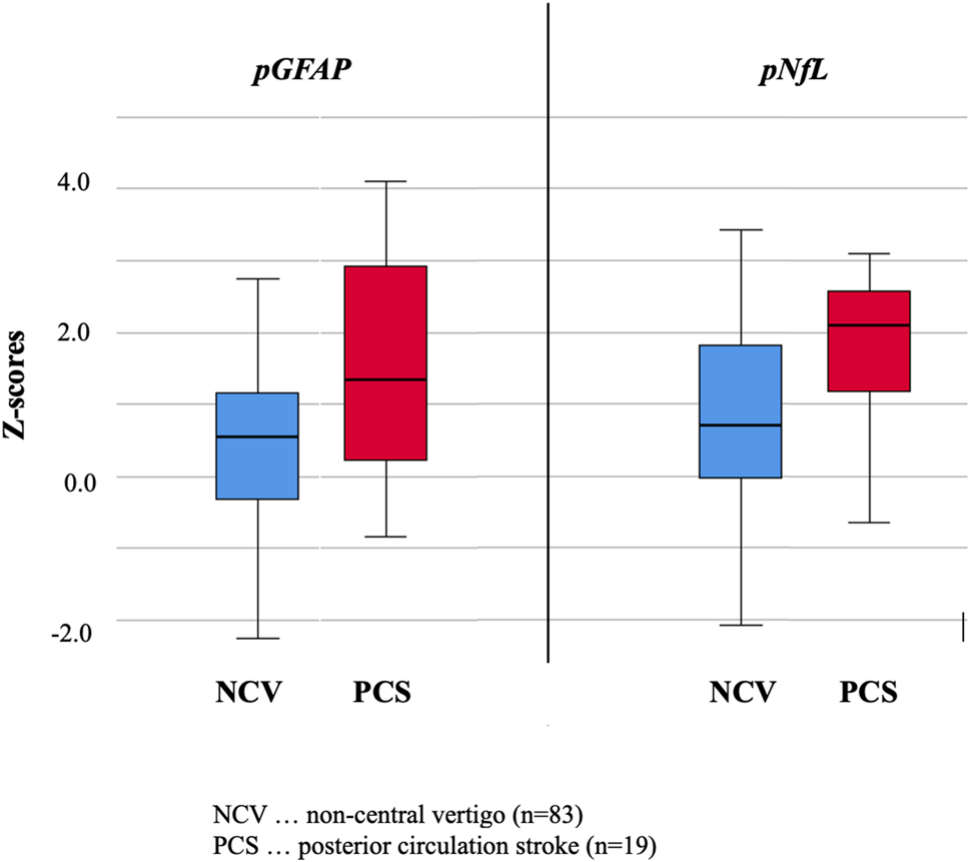
Differences of plasma glial fibrillary acidic protein (pGFAP) and neurofilament light chain (pNfL) Z-scores in non-central vertigo patients versus posterior circulation stroke patients

Absolute pNfL levels (median 21.0 pg/mL versus 12.4 pg/mL, *p* = 0.001) and median pNfL Z-scores (2.1 versus 0.7, *p* = 0.004, Table 1, Fig. 2) were higher in PCS compared to patients with non-central vertigo causes. The association of higher pNfL Z-scores in patients with PCS remained significant after adjusting the analysis for the time period of symptom onset to blood collection (*p* = 0.035). Various cut-off levels for pNfL Z-scores were evaluated (Table 2). A pNfL Z-score ≥ 1.0 was associated with PCS (OR 8.8, 95% CI 2.4–32.7, *p* < 0.001), yielding a sensitivity of 84.2%, a specificity of 62.4% and a negative predictive value of 94.6%. This association remained again robust after adjusting for symptom duration (OR 10.7, 95% CI 2.8–41.6, *p* < 0.001).

**Table 2:** Sensitivity and specificity analyses of different cut-off levels of neurofilament light chain and glial acidic fibrillary protein in plasma

	Central vertigo (*n* = 19)	Non-central vertigo (*n* = 83)	*P* value	Odds ratio (95% CI)	Sensitivity	Specificity	Negative predictive value	*P* value adjusted*	Odds ratio adjusted* (95% CI)
Neurofilament light chain									
Z-score ≥ 1.0	16 (84.2)	32 (37.6)	< 0.001	8.8 (2.4–32.7)	84.2%	62.4%	94.6%	< 0.001	10.7 (2.8–41.6)
Z-score ≥ 1.2	14 (73.7)	30 (35.3)	0.002	5.1 (1.70–15.6)	73.7%	64.7%	91.7%	0.002	6.5 (2.0–21.1)
Z-score ≥ 1.5	11 (57.9)	25 (29.4)	0.018	3.3 (1.2–9.2)	57.9%	70.6%	88.2%	0.005	5.1 (1.6–15.5)
Glial acidic fibrillary protein									
Z-score ≥ 1.0	11 (57.9)	24 (28.2)	0.013	3.5 (1.3–9.8)	57.9%	71.8%	88.4%	0.025	3.5 (1.2–10.3)
Z-score ≥ 1.2	10 (52.6)	20 (23.5)	0.011	3.6 (1.3–10.1)	52.6%	76.5%	87.8%	0.019	3.8 (1.2–11.3)
Z-score ≥ 1.5	7 (36.8)	11 (12.9)	0.013	3.9 (1.3–12.1)	36.8%	87.1%	86.0%	0.015	4.8 (1.4–17.1)
Combination of neurofilament light chain and glial acidic fibrillary protein									
Both Z-scores ≥ 1.0	11 (57.9)	16 (19.3)	< 0.001	5.8 (2.0–16.7)	57.9%	80.7%	89.3%	0.003	5.7 (1.8–17.7)

*adjusted for duration of vertigo symptoms

Absolute pGFAP levels were higher in PCS patients compared to patients with non-central vertigo (median 211.9 pg/mL versus 116.8 pg/mL, *p* < 0.001). The median pGFAP Z-scores were also significantly elevated in PCS patients (1.3 versus 0.6, *p* = 0.016, Table 1, Fig. 2). This association remained significant after adjusting the analysis for the time period of symptom onset to blood collection (*p* = 0.008). A pGFAP Z-score ≥ 1.0 was associated with PCS (OR 3.5, 95% CI 1.3–9.8; *p* = 0.013, Table 2), yielding a sensitivity of 57.9%, a specificity of 71.8% and a negative predictive value of 88.4%. This association remained also robust after adjusting for symptom duration (OR 3.5, 95% CI 1.2–10.3, *p* = 0.025).

Higher cut-off levels of pNfL or pGFAP Z-scores did not result in improved sensitivity, specificity or negative predictive value (Table 2).

When combining both biomarkers, defined as a pNfL Z-score ≥ 1.0 and a pGFAP Z-score ≥ 1.0, there was a significant association with PCS (OR 5.8, 95% CI 2.0–16.7, *p* < 0.001). This combination yielded a sensitivity of 57.9%, a specificity of 80.7% and a negative predictive value of 89.3% (Table 2) and remained robust after adjusting for symptom onset (OR 5.7, 95% CI 1.8–17.7, *p* = 0.003).

## Discussion

In this prospective observational study conducted in an emergency department setting, we could show that pNfL and pGFAP levels, particularly Z-scores of these biomarkers that adjust for age, sex and BMI, were significantly higher in patients with PCS than in non-central vertigo patients. Notably and not surprisingly, patients with non-central vertigo causes had a significantly longer symptom duration (0.3 versus 4.5 days); however, results remained robust after correction for symptom onset. Although the combination of pNfL and pGFAP enhanced specificity for PCS in our analysis, the sensitivity of this approach was rather moderate and the negative predictive value was lower compared to single pNfL analysis.

While there are no available studies yet that have investigated pNfL in vertigo or PCS patients, two studies investigating pGFAP in an acute vertigo cohort exist. These single studies did not show a significant difference in pGFAP levels in central versus non-central causes of vertigo, but were limited by less sensitive GFAP measurements using enzyme-linked immunosorbent assay (ELISA) based techniques [4, 6]. In the present study, pNfL and pGFAP levels were measured using the ultrasensitive SIMOA technique, that has also been used in prior studies investigating pNfL and pGFAP in acute ischemic stroke or intracranial hemorrhage [10, 11, 16].

Our results are supported by previous studies that revealed elevated NfL, a marker of axonal injury, early after brain tissue damage in ischemic stroke compared to healthy controls or stroke mimics [17–19]. Furthermore, blood GFAP, an astrocytic marker, has also been linked to brain tissue injury, especially in intracerebral hemorrhage [7], but was also elevated in patients with recent small subcortical infarction compared to healthy controls [11].

It is of note that NfL and GFAP are not disease-specific parameters as they indicate brain tissue damage irrespective of the underlying etiology, therefore, false positive results are possible in individuals with concomitant chronic neurological diseases that also involve neuron and glial cell damage [19–21]. Such patients were, however, excluded from our analysis.

The sensitivity and the negative predictive value for PCS was higher for pNfL than for pGFAP Z-scores in our analysis. This is an interesting, rather unexpected finding considering previous studies that have investigated time-dependent relationships of NfL and GFAP concentrations after ischemic stroke. These studies showed that GFAP reached the peak in serum/plasma earlier than NfL (day 1–3 versus day 7 post-stroke) and, therefore, suggest that GFAP might be a more promising biomarker for the (hyper)acute phase of ischemic stroke compared to NfL [22, 23]. However, there are currently no published studies that have considered infarct location or focused specifically on the time-dependent dynamics of neuroaxonal or astroglial injury biomarkers in the brain structures of the posterior cerebral circulation. The high cell density and the increased vulnerability to hypoxic injury, especially of Purkinje cells in the cerebellum, might explain a faster axonal degeneration and a more rapid elevation of pNfL levels in PCS [24, 25].

From a clinical point of view, implementing pNfL and pGFAP measurements in diagnostic pathways could improve early risk stratification in patients with acute vertigo. The use of blood biomarkers could be helpful in further patient selection for brain imaging (MRI) and, therefore, improve the distribution of resources in the emergency department. Patients with elevated pNfL and pGFAP levels may warrant further MRI neuroimaging and/or hospital admission, whereas those with low levels could potentially be managed on an outpatient basis. Thus, the number of unnecessary imaging, other diagnostic tests and hospital stays could be potentially reduced. This approach could be particularly valuable in interdisciplinary emergency departments, where accurate triage between different medical specialties is important. However, we acknowledge that further confirmation in larger, multicentre studies is necessary.

Our study population was recruited from an acute neurological emergency service within an interdisciplinary emergency centre with a clinical selection approach and is, therefore, representing a real-world cohort of unselected vertigo patients. Despite that, patients received a thorough diagnostic work-up with a complete neurological work-up including neuroimaging in all cases.

Our study also has several limitations. The most important is the low sample size and single-centre design that limits the generalizability of our findings. In addition, the patient cohort was quite heterogenous as we decided not to exclude patients with non-vestibular or unspecific vertigo in order to represent a real-world vertigo cohort in an interdisciplinary emergency department. Furthermore, it is known that small brainstem infarctions might not be detected on early brain MRI in the first 24 h after stroke onset. [3] Thus, patients with non-central vertigo causes and initial DWI-negative MRI could have possibly been false-negative, which has to be mentioned as a limitation in our study. However, as non-central vertigo patients, especially patients with unspecific vertigo, received further diagnostic tests including neurological examination and consultation of other medical specialties (including otorhinolaryngology) to identify the final vertigo diagnosis, this seems rather unlikely.

Finally, further validation in larger, multicentre cohorts also in comparison to healthy control populations is necessary to further investigate this diagnostic measure and to establish a valid cut-off level for pNfL and pGFAP Z-scores before implementation in routine clinical practice can be considered.

In conclusion, pNfL and pGFAP show promise as useful biomarkers for identifying PCS in acute vertigo patients in the emergency department. In addition to clinical assessment and imaging, the use of these blood biomarkers could support clinical decision-making and allocation of resources in this setting.

## Abbreviations

CT: Computed tomography
DWI: Diffusion weighted imaging
GFAP: Glial fibrillary acidic protein
MRI: Magnetic resonance imaging
NfL: Neurofilament light chain
PCS: Posterior circulation stroke
pGFAP: Plasma glial fibrillary acidic protein
pNfL: Plasma neurofilament light chain
SIMOA: Single Molecule Array
